# Low predictive power of clinical features for relapse prediction after antidepressant discontinuation in a naturalistic setting

**DOI:** 10.1038/s41598-022-13893-9

**Published:** 2022-07-01

**Authors:** Isabel M. Berwian, Julia G. Wenzel, Leonie Kuehn, Inga Schnuerer, Erich Seifritz, Klaas E. Stephan, Henrik Walter, Quentin J. M. Huys

**Affiliations:** 1 Princeton Neuroscience Institute, Princeton University, Princeton, USA; 2 Translational Neuromodeling Unit, University of Zurich and ETH Zurich, Zurich, Switzerland; 3 Charité Universitätsmedizin, Campus Charité Mitte, Berlin, Germany; 4 Department of Psychiatry, Psychotherapy and Psychosomatics, Psychiatric Hospital, University of Zurich, Zurich, Switzerland; 5 Wellcome Centre for Human Neuroimaging, University College London, London, UK; 6 Max Planck Institute for Metabolism Research, Cologne, Germany; 7 Division of Psychiatry and Max Planck Centre for Computational Psychiatry and Ageing Research, University College London, London, UK; 8 Camden and Islington NHS Foundation Trust, London, UK

**Keywords:** Risk factors, Psychology

## Abstract

The risk of relapse after antidepressant medication (ADM) discontinuation is high. Predictors of relapse could guide clinical decision-making, but are yet to be established. We assessed demographic and clinical variables in a longitudinal observational study before antidepressant discontinuation. State-dependent variables were re-assessed either after discontinuation or before discontinuation after a waiting period. Relapse was assessed during 6 months after discontinuation. We applied logistic general linear models in combination with least absolute shrinkage and selection operator and elastic nets to avoid overfitting in order to identify predictors of relapse and estimated their generalisability using cross-validation. The final sample included 104 patients (age: 34.86 (11.1), 77% female) and 57 healthy controls (age: 34.12 (10.6), 70% female). 36% of the patients experienced a relapse. Treatment by a general practitioner increased the risk of relapse. Although within-sample statistical analyses suggested reasonable sensitivity and specificity, out-of-sample prediction of relapse was at chance level. Residual symptoms increased with discontinuation, but did not relate to relapse. Demographic and standard clinical variables appear to carry little predictive power and therefore are of limited use for patients and clinicians in guiding clinical decision-making.

## Introduction

Depressive disorders are a major burden to societies worldwide, being the single largest contributors to years lived with disability^1^. This is largely due to the often chronic relapsing nature of depression, which underlies the functional and social impairments it brings^2^. Hence, successful treatment of a particular depressive episode, e.g. by achieving response to an antidepressant medication (ADM), is critical, but only the first step.

Thus, the prevention of relapses is the next step as over half of the patients with one depressive episode will experience a second one and the risk of relapse only increases further thereafter^3^. Preventing relapses is of paramount importance for the longer-term course of the illness, and a number of strategies exist. One important strategy is continuation and maintenance treatment with ADM, which reduces the risk of relapse^4–7^. However, there is still a risk of breakthrough depression, i.e. the development of further depressive episodes while taking ADMs^8^. Furthermore, many patients want to discontinue their ADM due to side-effects such as weight gain and sexual dysfunction^9^ or adhere only partially^10^. At the same time, one in three patients relapses within 6 months after discontinuation^4^.

Thus, not all patients benefit equally from continuation treatment and there appears to be variation in individual trajectories after the initial response to ADMs^11–14^. Markers that identify these trajectories and separate those patients who can safely discontinue their ADMs from those with a higher risk of relapse after discontinuation clearly have the potential of improving this situation.

Indeed, current guidelines take some of this variation into account, and recommend continuation treatment for 4–9 months after the first depressive episode and 2 years or more after recurrent episodes^15,16^. More recently, guidelines also refer to residual symptoms and physical and psychological comorbidities^17^. These recommendations are based on evidence derived from the natural course of depression and overall relapse risk^18–20^. However, the importance of these markers has been disputed^21^: two meta-analyses came to diametrically opposed conclusions about the relevance of the number of prior episodes^5,22^, and five separate meta-analyses have failed to find an effect of length of ADM treatment on relapse risk after discontinuation^4–6,22,23^.

Several other predictors of relapse after discontinuation exist in the literature^21^. These include ethnicity^24^, neurovegetative symptoms^25^, melancholic subtype^25^, anxiety^26^, somatic pain^27^ and response pattern to drug^28,29^. Only the last predictor, having a placebo drug response, i.e. fast but unstable response, compared to a true drug response, i.e. slower but sustained response^30^, has been replicated^28,29^. Unfortunately, assessing this measure will be difficult in clinical practice.

Two further points complicate this picture. The first point relates to a methodological problem. Studies have usually mainly focused on asking whether a particular variable differs between groups of patients who do and do not go on to relapse. Unfortunately, while such differences might reach statistical significance, they might still fail to perform well as predictors^31^. Regression analyses have been used in some studies, but using a simple regression bears the risk of overfitting^32^. To make inferences about a new patient in a practice, the predictive power in cases outside of the sample used to fit the regression model needs to be determined, e.g., using cross-validation. To our knowledge, this has not been reported in the literature so far. Second, most studies have been performed in the setting of double-blind RCTs. While this is the ideal approach to examine whether an active compound has a causal role in reducing relapse, it may underestimate relapse rates after discontinuation because medication discontinuation might have psychological effects in addition to direct pharmacological effects.

Here, we report findings from the AIDA study—a two-centre, longitudinal, naturalistic observational study of antidepressant discontinuation. Our first aim was to investigate the extent to which variables which are easily assessable in a naturalistic setting can predict individual relapse risk and possibly guide the decision to discontinue or not. We paid specific attention to previously reported clinical predictors and examined their performance in a naturalistic setting. A secondary goal of this study was to understand the effects of discontinuation itself and how these relate to relapse. Accordingly, we investigated if any of the state-dependent variables changed with discontinuation and if that change differed between relapsers and non-relapsers.

## Methods and material

### Participants

We recruited patients who decided to discontinue their medication independently from study participation after they were diagnosed with Major Depressive Disorder (MDD) and had (a) experienced one severe^33^ or multiple depressive episodes, (b) initiated antidepressant treatment during the last depressive episode and (c) now achieved stable remission, i.e. a score of less than 7 on the Hamilton Depression Rating Scale 17^34^ for 30 days. To identify disease and medication effects, we also recruited healthy controls (HC) matched for age, sex and education. See Section S1.1 for detailed inclusion and exclusion criteria. All participants gave informed written consent and received monetary compensation for the time of participation. Ethical approval for the study was obtained from the cantonal ethics commission Zurich (BASEC: PB_2016-0.01032; KEK-ZH: 2014-0355) and the ethics commission at the Campus Charité-Mitte (EA 1/142/14), and procedures were in accordance with the Declaration of Helsinki.

### Study design

**Figure 1:**
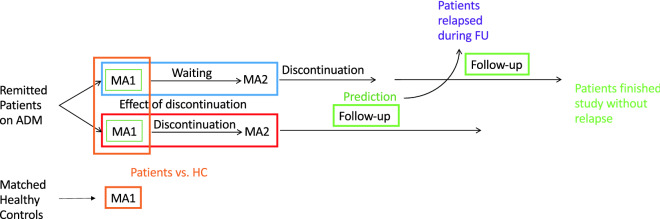
Study design: we recruited remitted, medicated patients on antidepressant medication (ADM) and matched healthy controls (HC). They were assessed and compared at main assessment 1 (MA1) to identify traits characterising the remitted, medicated state. Next, patients were randomised to either discontinue their medication before MA2 (bottom arm, “discontinuation group” or enter a waiting period while continuing their ADM matched to the length of discontinuation time (top arm, “waiting group”). Differences in changes between MA1 and MA2 in the two separate groups were investigated to gain an understanding of the effects underlying discontinuation. Patients in the waiting group discontinued their ADM after MA2. After discontinuation, all patients entered the follow-up (FU) period of 6 months, whereas some patients had a relapse during this period and some patients finished this period without relapse. Differences in characteristics at MA1 of patients who relapsed and patients who did not relapse during FU provide information on which variables relates to relapse risk and can be used to identify predictors of relapse after ADM discontinuation.

The study design is depicted in Fig. 1. Trained staff interviewed remitted patients on ADM to assess in- and exclusion criteria during a baseline assessment (BA). The BA consisted of the assessment of current symptoms and present and past diagnoses, as well as a short neuropsychological testing and a questionnaire batch assessing stable traits. Patients meeting inclusion criteria were randomised to one of two study arms. Of note, the first 10 participants at each site were all assigned to arm 1D2 (1/2 represents the number of the main assessment, “D” represents discontinuation). Participants in arm 1D2 underwent the first main assessment (MA1) including a questionnaire assessing state variables, then gradually discontinued their medication over up to 18 weeks and then underwent a second main assessment (MA2). Participants in arm 12D underwent both main assessments before discontinuation. During discontinuation all patients were contacted every 2 weeks for a telephone assessment. This two-arm design allowed us to identify discontinuation effects while controlling for time, learning and repetition effects. After discontinuation, all patients entered a follow-up period of 6 months. During that period, they were contacted for telephone assessments at weeks 1, 2, 4, 6, 8, 12, 16 and 21 to assess relapse status. If telephone assessment indicated a possible relapse, patients were invited to an on-site structured clinical interview (SCID-I^35^) to assess criteria for relapse, i.e. fulfilling the diagnosis of a depressive episode according to the Diagnostic and Statistical Manual of Mental Disorders (4th ed., text rev.; DSM-IV-TR^3^). If these criteria were fulfilled, they underwent a final assessment (FA). If no relapse occurred, the FA took place in week 26. HC underwent MA1 only. In addition to the measures reported here, participants also underwent functional magnetic resonance imaging, a range of behavioural task, electroencephalography and blood sampling during the main assessments. See supplementary Section S1.2 for detailed procedures of the assessment sessions and S1.3 for observer-rated and self-report measures. Participant recruitment took place between July 2015 and January 2018.

### Measures

We included 18 measures spanning four categories: demographics, current symptoms, clinical history and treatment. Measures were chosen based on two criteria: (1) they have previously been related to relapse after antidepressant discontinuation^21^, and (2) they can easily be assessed during a routine clinical visit, do not require extensive training or equipment and have a plausible relation to relapse risk. Individual measures in each category are listed in Table 1 and described in supplementary Section S1.3. Ten of these variables were previously investigated in randomised controlled trials (RCTs). All listed measures can be assessed before discontinuation and will be included in the prediction analysis. We additionally compared discontinuation time between relapsers and non-relapsers, but did not include it in the prediction model. All measures from the category current symptoms were re-assessed at MA2.

**Table 1:** Participant characteristics and complete-case analyses.

	Patients vs. HC			Relapse vs. No relapse			Full GLM		Regularised GLM
	Patients (n = 104)	HC (n = 57)	p value	Relapse (n = 30)	No relapse (n = 54)	p value	Coefficients	p value	Coefficients
**Demographics**									
Age	34.85 (11.10)	34.12 (10.95)	0.69	37.00 (10.95)	33.56 (11.23)	0.18	0.073	0.91	0
Male sex, No. (%)	24 (23)	17 (30)	0.35	7 (23)	13 (24)	0.94	− 0.08	0.77	0
Intelligence$^{\mathrm{b}}$	28.12 (4.43)	27.79 (4.13)	0.65	29.05 (3.76)	27.8 (4.64)	0.09	− 0.38	0.31	− 0.18
Site Berlin, No. (%)	28 (27)	22 (39)	0.13	9 (30)	14 (35)	0.69	0.4426	0.20	0
**Clinical predictors**									
Current symptoms									
Residual depression$^{\mathrm{b}}$	3.73 (3.82)	0.77 (1.25)	< 0.001	3.77 (5.23)	3.15 (2.59)	0.47	0.15	0.65	0
Residual anxiety$^{\mathrm{b}}$	2.89 (2.34)	1.63 (1.76)	< 0.001	3.03 (3.02)	2.56 (2.01)	0.39	0.61	0.20	0
Somatic pain$^{\mathrm{b}}$	0.32 (0.23)	0.15 (0.26)	< 0.001	0.39 (0.23)	0.28 (0.22)	0.042	− 0.63	0.08	− 0.27
General impairment$^{\mathrm{b}}$	0.31 (0.25)	0.12 (0.18)	< 0.001	0.35 (0.31)	0.25 (0.19)	0.07	− 0.87	0.13	− 0.06
Clinical history									
Age of onset	–	–	–	25.00 (9.88)	23.8 (8.34)	0.56	0.07	0.91	0
Chronicity$^{\mathrm{b}}$	–	–	–	8.07 (10.44)	8.15 (9.42)	0.97	0.35	0.30	0
Severity$^{\mathrm{b}}$	–	–	–	6.97 (1.13)	7.04 (1.30)	0.80	0.19	0.56	0
Number of prior episodes	–	–	–	2.77 (1.79)	2.28 (1.48)	0.18	− 0.42	0.58	0
Severity factor$^{\mathrm{c}}$	–	–	–	0.09 (0.38)	− 0.03 (0.33)	0.15	0.20	0.84	− 0.32
Comorbidities$^{\mathrm{b}}$	–	–	–	0.70 (1.02)	0.80 (1.17)	0.71	0.08	0.81	0
Treatment									
Treated by GP only, No. (%)	–	–	–	10 (33)	8 (15)	0.048	− 0.94	0.0051	− 0.29
Duration of ADM intake$^{\mathrm{c}}$	–	–	–	24 (29)	22.5 (38)	0.66	− 0.13	0.72	0
Medication load$^{\mathrm{c}}$	–	–	–	0.0068 (0.0041)	0.008 (0.004)	0.26	0.15	0.62	0
Psychotherapy$^{\mathrm{c}}$	–	–	–	0.38 (0.39)	0.39 (0.40)	0.95	− 0.54	0.12	0
Tapering in days	–	–	–	51.10 (40.64)	48.89 (39.79)	0.81	–	–	–

$^\mathrm{a}$ Unless stated otherwise, mean (SD) are shown; $^\mathrm{b}$Determined as follows: intelligence: Mehrfachwahl Wortschatz Test^38^; residual depression: Inventory of Depressive Symptomatology-Clinician Rated^39^; residual anxiety: screening generalised anxiety disorder (GAD-7^40^); somatic pain: somatisation subscale of symptom checklist 90 (SCL-90^41^); general impairment: global severity index of SCL-90; chronicity: numbers of months sick within the last 5 years; severity: symptoms during the last episode; comorbidities: number of past and present psychiatric diagnoses; $^\mathrm{c}$Computation of the variables is described in the Section S1.4; *GP* general practitioner, *ADM* antidepressant medication, *HC* healthy controls, *GLM* general linear model. Intercept for regression models not shown.

### Data analysis

Analyses were performed using Matlab version 9.1.0.441655 (R2016b) according to our a priori analysis plan available at https://gitlab.ethz.ch/tnu/analysis-plans/aidaz_analysis_plan_clinical_prediction.

#### Association analyses

Candidate predictor variables were first identified by assessing group differences between patients and HC and between relapsers and non-relapsers. Two-sample two-tailed independent t-tests were used for continuous and chi-squared tests for categorical variables (including psychotherapy). We report results using no multiple comparison correction, i.e. considering tests to be significant at p < 0.05 and indicate if they survive correction using false discovery rate (FDR). The former allows for better interpretation of non-significant findings. The latter helps to control for the number of tests we applied since we are investigating a range of variables increasing the risk of false positives. In contrast to Bonferroni correction, FDR-based corrections do not make the assumption that tests are independent.

Complete-case analyses can yield biased results. We therefore examined whether patients who dropped out differed from patients who finished the study. For this, we repeated the above analyses procedure comparing patients who finished the study and patients who dropped out after MA1. We next performed Cox proportional hazards regression models, relating predictor variables to time to relapse or dropout. For these analyses, all variables were mean-centered and normalised. We first performed this for each measure individually and then included all measures in the same Cox regression, to compare predictors. Since our goal is to predict relapse after antidepressant discontinuation, we performed the latter analysis first for the time after discontinuation, but repeated the analysis by extending the observation period to include the time of discontinuation. To test the assumption of proportional hazards, we conducted the Schoenfield individual and global test for each individual predictor variable using the scaled Schoenfield residuals and visually inspected the plots of the residuals to exclude an association of the residuals with time.

#### Prediction analyses

To examine whether clinical variables have predictive value, we first fitted a full logistic general linear model (GLM) including all relapsers and non-relapsers to determine which variables made a significant contribution to the prediction, the total variance that can be explained by the combined predictors, the area under the curve, the best threshold as well as the sensitivity and specificity at this threshold.

However, as there are 18 predictors for 84 data points any results for the current sample may generalise poorly due to overfitting. To address the high number of predictors compared to the small sample size, we used an elastic net with both an L1 and L2 regularisation^36^ as implemented by the lassoglm function in Matlab. We applied tenfold cross validation with stratification to optimize strength of the L1-regularisation parameter ($\lambda$). This was repeated for a range of $\alpha$ values and the optimum was chosen.

Next we repeated this entire procedure within a nested cross-validation procedure to examine generalisation to data not seen by the algorithm. The outer loop consisted of a leave-one-out cross-validation (LOOCV). One subject was first set aside, then the full GLM or the regularised GLM, respectively, was fitted to all other subjects. Then, the group membership of the left-out subject was predicted using parameter estimates (regression weights) obtained from the other subjects. The classification threshold was set to 0.5. These predictions were used to compute the balanced accuracy and the probability that these predictions would not be better than chance was determined with a binomial test. To determine receiver operating curves for left out subjects, we categorised these subjects as relapsers or non-relapsers for varying thresholds and computed how many subjects were categorised correctly for each threshold.

#### Discontinuation analyses

To investigate the discontinuation effect and the interaction between discontinuation and relapse, we applied mixed analyses of variance (ANOVAs) with group (1D2 vs. 12D) and (relapse vs. no relapse in the discontinuation group, i.e. patients who discontinued before MA2, only) as between-subjects factor and time (MA1 vs. MA2) as within-subject factor.

#### Exploratory analyses

Due to new findings that quality of life relates to relapse^37^ and to ensure that our results are not limited to our set of pre-selected variables described in our analysis plan, we run exploratory analyses comparing quality of life, income, psychiatric family history, alcohol consumption and smoking between patients who would go on to relapse and those who remained well.

## Results

### Participants

Nineteen (15%) of 123 included patients dropped out of the study prior to the first main assessment and were not further analysed. Of the 104 who completed the first main assessment (28 recruited in Berlin and 76 recruited in Zuerich), 91 (88%) completed both main assessments (44 off medication after discontinuation in arm 1D2 and 47 on medication prior to discontinuation in arm 12D). Mean (standard deviation) number of days between first and second main assessment was 72 (40) for group 1D2 and 55 (36) in group 12D (t(85) = 2.02, p = 0.046). Of the 91 patients who completed both main assessments, 89 (86%) achieved antidepressant discontinuation and 83 (67%) reached a study endpoint by either remaining in remission for 6 months, or only restarting antidepressants after reaching criteria for relapse. One additional patient was categorised as relapser after meeting criteria for relapse for 10 days (shorter than the length criterion of 14 days) and quick improvement after treatment re-initiation. Of these 84 patients, 30 (36%) had a relapse during the follow-up period. Detailed reasons for dropouts are depicted in Fig. S1.

### Association analyses

#### Complete-case analysis

Patients and healthy controls (n = 57) were matched for demographic variables but patients had elevated residual depression (t(159) = 5.68, p < 0.001, CI = 1.93–3.99), anxiety (t(159) = 3.56, p < 0.001, CI = 0.56–1.96) and somatic pain symptoms (t(159) = 4.47, p < 0.001, CI = 0.098–0.254) and scored higher on general impairment (t(159) = 5.02, p < 0.001, CI = 0.11–0.26; Table 1). These results survived correction for multiple comparison.

**Table 2:** Intention-to-treat analyses.

	CR for each variable independently		CR incl. all variables		CR after ADM reduction incl. all variables	
	Coefficents	p value	Coefficents	p value	Coefficents	p value
**Demographics**						
Age	0.19	0.28	− 0.22	0.61	− 0.12	0.77
Male sex, No. (%)	0.03	0.87	0.23	0.29	0.18	0.38
Intelligence$^{\mathrm{b}}$	0.29	0.13	0.28	0.34	0.18	0.51
Site Berlin, No. (%)	− 0.04	0.84	− 0.37	0.16	− 0.19	0.41
**Clinical predictors**						
Current symptoms						
Residual depression$^{\mathrm{b}}$	0.19	0.37	0.08	0.79	− 0.03	0.92
Residual anxiety$^{\mathrm{b}}$	0.21	0.24	− 0.27	0.39	− 0.20	0.51
Somatic pain$^{\mathrm{b}}$	0.31	0.08	0.44	0.08	0.22	0.32
General impairment$^{\mathrm{b}}$	0.32	0.04	0.33	0.36	0.30	0.38
Clinical history						
Age of onset	0.05	0.78	0.06	0.89	0.01	0.98
Chronicity$^{\mathrm{b}}$	− 0.01	0.94	− 0.29	0.32	− 0.22	0.40
Severity$^{\mathrm{b}}$	− 0.04	0.83	− 0.07	0.77	0.14	0.54
Number of prior episodes	0.18	0.21	0.13	0.80	− 0.05	0.92
Severity factor$^{\mathrm{c}}$	0.25	0.13	0.10	0.89	0.21	0.76
Comorbidities$^{\mathrm{b}}$	− 0.03	0.88	− 0.08	0.76	− 0.08	0.73
Treatment						
Treated by GP only, No. (%)	0.36	0.03	0.66	0.005	0.41	0.043
Length of ADM intake	− 0.25	0.22	− 0.06	0.84	− 0.07	0.80
Medication load$^{\mathrm{c}}$	− 0.25	0.22	− 0.15	0.51	− 0.03	0.88
Psychotherapy$^{\mathrm{c}}$	0.01	0.96	0.34	0.15	0.06	0.77

$^\mathrm{a}$Unless stated otherwise, mean (SD) are shown; $^\mathrm{b}$Determined as follows: intelligence: Mehrfachwahl Wortschatz Test^38^; residual depression: Inventory of Depressive Symptomatology-Clinician Rated^39^; residual anxiety: screening generalised anxiety disorder (GAD-7^40^); somatic pain: somatisation subscale of symptom checklist 90 (SCL-90^41^) general impairment: global severity index of SCL-90; chronicity: numbers of months sick within the last 5 years; severity: symptoms during the last episode; comorbidities: number of past and present psychiatric diagnoses; $^\mathrm{c}$Computation of the variables is described in the Section S1.4. *GP* general practitioner, *ADM* antidepressant medication, *CR* cox regression, *FU* follow-up period (up to 6 months from end of discontinuation).

**Figure 2:**
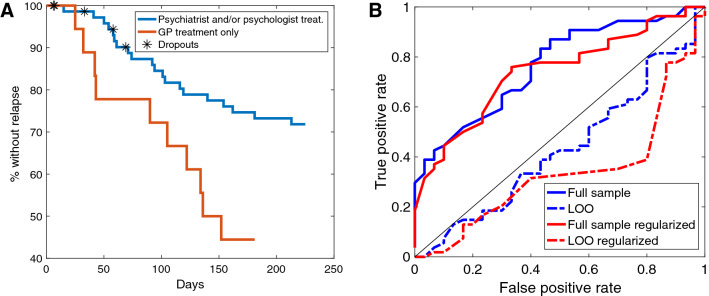
(**A**) Survival curves for time until relapse during follow-up period for patients who were only treated by a general practitioner (GP) or additionally by a psychiatrist or psychologist. (**B**) Prediction: Receiver operating curves for a standard general linear model (blue) and a regularised general linear model using least absolute shrinkage and selection operator and elastic net (red) using the full sample (solid lines) and for subjects left out of the fit using leave-one-out (LOO) cross-validation (dashed lines).

We first performed a complete-case analysis on the 84 patients who either reached the follow-up period without relapse or relapsed during that period to maximise the chances of identifying potentially predictive variables. Patients who went on to relapse after ADM discontinuation had increased somatic pain (t(82) = 2.07, p = 0.042, CI = 0.004–0.21) and were more often treated by a general practitioner only rather than a psychiatrist ($\chi ^2$ = 3.93, p = 0.048), though these differences did not survive correction for multiple comparisons (Table 1). To assess the unique contributions of the predictor variables, all measures were combined in a single multiple regression model. This revealed treatment by GP only as the sole significant variable associated with relapse (b = − 0.94, p = 0.005; Table 1). Of the 30 relapsers, 10 were treated by a GP only, while only 8 of 54 non-relapsers were treated by a GP only.

Complete-case analyses may yield biased results. Patients who dropped out had more residual symptoms (t(102) = − 2.01, CI: − 3.73 to − 0.025, p = 0.047) and more symptoms during the last episode (t(102) = − 2.09, CI: − 1.24 to − 0.033, p = 0.039) (Table S1). These differences did not survive correction for multiple comparisons.

#### Intention-to-treat analyses

As there were differences between patients who completed the study and those who dropped out, we performed intention-to-treat analyses using Cox proportional hazards including all the 89 patients who completed discontinuation. The Schoenfield tests for each predictor variable were all non-significant, indicating that the assumptions for the cox proportional hazard models were met. These results were further confirmed by visual inspection indicating no association of time with the scaled Schoenfield residuals. The Cox proportional hazard models revealed that general impairment (b = 0.32, p = 0.044, CI = 0.008–0.632) and treatment by GP only (b = 0.36, p = 0.025, CI = 0.045–0.666) were significantly associated with shorter time to relapse, though neither survived correction for multiple comparisons. Of note, no effect was found for the current symptoms and symptoms during the last episode which distinguished patients who dropped out (Table 2). To assess the unique contributions of the predictor variables, all measures were combined in a Cox multiple regression model. This again revealed treatment by GP only as the uniquely significant predictor (b = 0.662, p = 0.005; Table 2, Fig. 2A). GP only treatment was also the only variable associated with shorter time to relapse in an extended intention-to-treat analysis including an additional 6 patients who initiated but did not complete antidepressant discontinuation (Table 2).

### Prediction of relapse

To ascertain whether these findings could inform clinical practice, we next assessed how well clinical variables were able to predict relapses. Individual predictions could only meaningfully be assessed on the complete-case data. The multiple linear regression with all variables included achieved an area under the curve (AUC) of 0.76 with a sensitivity of 0.87 and specificity of 0.53 at the best cut-off (Fig. 2B). The model explained 21% of the variance. Such a performance is suggestive of clinical utility. However, with 18 predictor variables for 84 outcomes, this model may have overfitted the data and therefore may not generalize to new data.

We first examined overfitting through regularization via an elastic net, which pushes regression weights towards zero except for those predictor variables with most predictive power^36^. A standard approach with elastic nets, namely setting $\lambda$ to be one standard error larger than the value minimizing deviance, resulted in all regression weights being set to zero. A less stringent regularization using the value of $\lambda$ that minimized deviance resulted in a model with non-zero weights for five variables only (intelligence, somatic pain, general impairment, severity factor and treatment by GP only; Table 1) with an AUC of 0.74, a specificity of 0.66 and a sensitivity of 0.76 at the best cut-off value (Fig. 2B). Thus, five variables may suffice to predict relapse. However, since this is a within-sample analysis, it is still not clear whether and how well this result would generalise.

To determine how the models’ performances might generalise to new incoming patients, we approximated out-of-sample predictive accuracy using leave-one-out cross-validation (LOOCV). Doing this without regularisation yielded a balanced accuracy of 0.47. With regularisation, the balanced accuracy was 0.49. Neither prediction exceeded chance.

**Figure 3:**
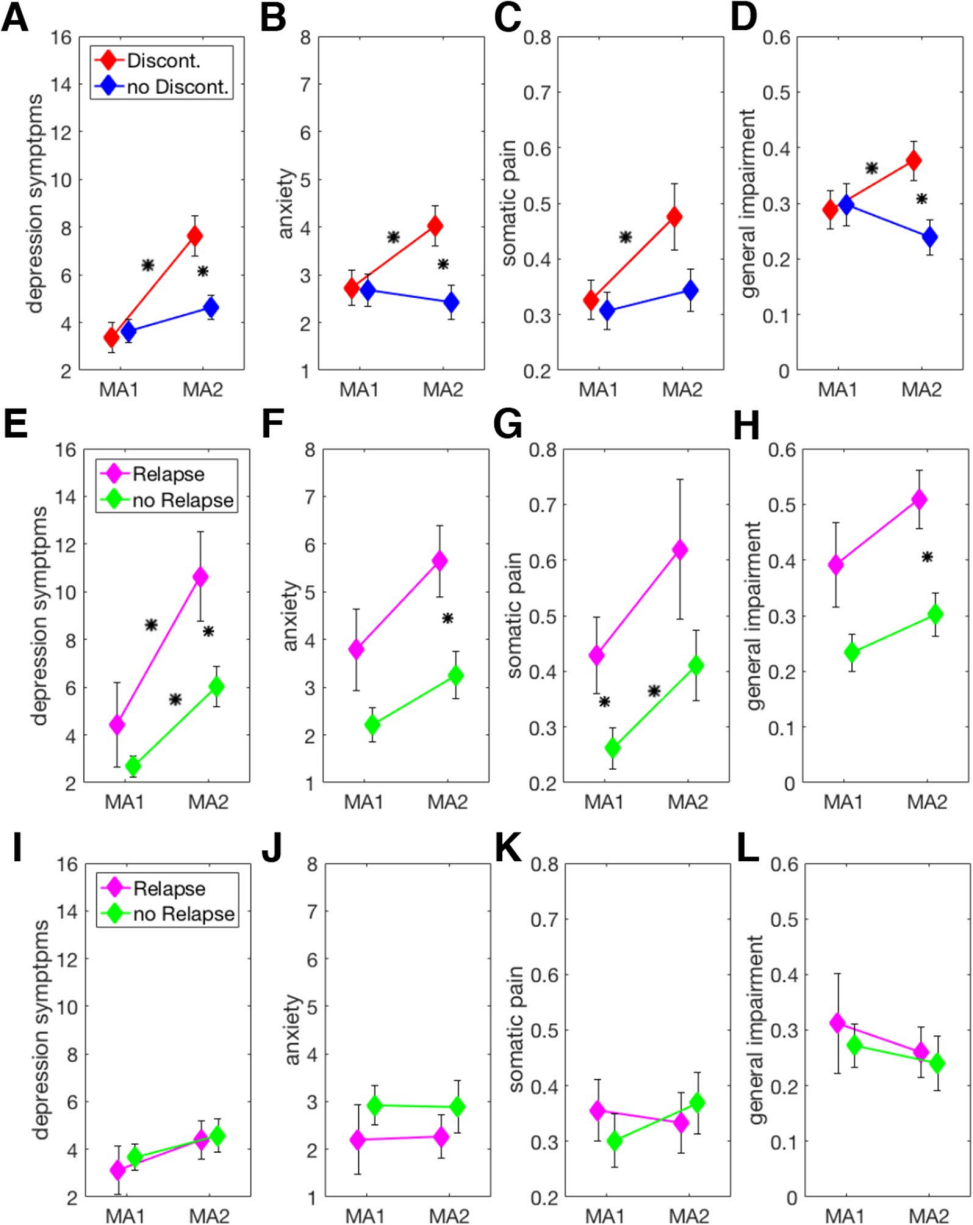
(**A**–**D**) Discontinuation effects: changes in symptoms from main assessment one (MA1) to main assessment two (MA2) for depression (**A**), anxiety (**B**), somatic pain (**C**) and general impairment (**D**) in patients who discontinued between the two assessments and patients who did not discontinue. (**E**–**H**) Discontinuation relapse interaction effects: Changes in symptoms from MA1 to MA2 for depression (**E**), anxiety (**F**), somatic pain (**G**) and general impairment (**H**) in patients who discontinued and either relapsed or remained well during the follow-up period. (**I**–**L**) Test–retest reliability for symptom measures: Changes in symptoms from MA1 to MA2 for depression (**I**), anxiety (**J**), somatic pain (**K**) and general impairment (**L**) in patients who did not discontinue and either relapsed or remained well during the follow-up period. Asterisks indicate a significant difference at p < 0.05 for FDR-corrected p values. Asterisks on top of a line relate to a within-subjects difference between MA1 and MA2 for the group indicated by the line. Asterisks between two lines relate to a between-subjects difference at the indicated time point.

### Discontinuation effect

The impact of antidepressant discontinuation on symptoms was examined by comparing changes in symptoms between the two main assessments in individuals randomized to groups 1D2 and 12D (Fig. 1). Discontinuation resulted in changes in residual symptoms in all four domains, including anxiety (F(1,89) = 6.55, p = 0.012), depression (F(1,89) = 1.46, p = 0.001) and general impairment (F(1,89) = 9.99, p = 0.002; Fig. 3A–D and Table S2). Post-hoc tests corrected for multiple comparisons with FDR indicated that no difference between groups exist at MA1, but did at MA2 and that the changes were due to an increase in symptoms in the group that discontinued their ADMs. For somatic pain, the interaction effect only showed a trend towards a significant difference (F(1,89) = 3.31, p = 0.072), and post-hoc tests of change only survived FDR correction in the discontinuation group. Excluding four patients from the analyses who received fluoxetine known to have long half-lives of 120 hours did not change the pattern or results.

### Association between discontinuation effect and relapse

We next asked whether the early effect of antidepressant discontinuation is associated with the ultimate risk of relapse. There was no interaction between the change in clinical measures before and after discontinuation (i.e. between the two main assessments in patients who discontinued before MA2, group 1D2; c.f. Fig. 1) and relapse (all p > 0.05). Instead, the analysis revealed main effects of relapse across all domains [anxiety (F(1,40) = 8.751, p = 0.005), general impairment (F(1,40) = 11.001, p = 0.003), depression (F(1,40) = 5.615, p = 0.023) and somatic pain (F(1,40) = 4.709, p = 0.036)]. Relapsers in the group 1D2 had more symptoms before starting discontinuation and symptoms in both relapsers and non-relapsers increased after discontinuation to a similar extent (Fig. 3E–H), while there were no changes in the group that did not discontinue before MA2 (i.e. group 12D; Fig. 3I–L, Table S2).

### Exploratory results

Quality of life did not differ between subsequent relapsers and non-relapsers (t(82) = − 1.35, p = 0.18) nor was it associated with time to relapse in a cox regression (b = − 0.28, p = 0.12). Similar results were obtained for income (t(82) = 0.57, p = 0.57; b = 0.9, p = 0.59), education (t(77) = − 0.38, p = 0.7; b = − 0.4, p = 0.83), family history (t(82) = − 0.27, p = 0.79; b = − 0.09, p = 0.65), smoking (t(82) = − 1.32 , p = 0.19; b = − 0.22, p = 0.25) and alcohol (t(82) = − 1.18, p = 0.24; b = − 0.21, p = 0.28). The inclusion of the latter variables into our out-of-sample prediction analyses also did not improve the predictive accuracy of the model (balanced accuracy: 0.5).

## Discussion

Antidepressant medications are efficient in the prevention of relapses and relapse rates after discontinuation are high^4^. Relapse rates in our study were also high, with one in three patients suffering a relapse within 6 months of discontinuation. This high relapse rate was observed even though the median duration of treatment was around 2 years, and hence at least as long as the duration of treatment recommended for recurrent illness^15,17^, and despite including only fully remitted patients with HAMD$^{\mathrm{17}}$ scores below 7. Relapses are not only important because they represent a period of renewed illness, but because any one episode has a 5-10% risk of becoming chronic^42^ and because early on in the disease additional episodes may mark the transition between those with a benign outcome and few lifetime episodes, and those with a malignant outcome and high risk of relapses^43–46^. This situation makes it evident that there is a clinical need to establish predictors of relapses specifically after antidepressant discontinuation, as such predictors could guide the discontinuation decision and in that way help reduce relapses and possibly even modify the long-term course of the illness.

A first pertinent step is the examination of the predictive power of clinical variables that are easily assessed in clinical practice. Our results suggest that such standard clinical variables carry at best weak predictive power. This conclusion relies on an examination of the likely generalisability of the associations. The approach is motivated by machine-learning approaches^32^. Rather than asking how well a set of variables can predict a particular outcome *within* a given dataset, the prediction is assessed on out-of-sample data *not used* in ascertaining the prediction parameters. Such approaches are standard in the field of machine-learning, and are becoming more prominent in neuroscience and psychiatry (e.g.^47–49^). We note that our cross-validation approach is not perfect as establishing a valid clinical predictor would ideally involve a fully independent dataset, but in our case this analysis indicates that the standard regression results do not carry predictive power.

Several aspects of the results from the standard approach are nevertheless noteworthy. First, in the full regression model and the intention-to-treat analyses including all predictors, only GP only treatment emerged as significantly associated with relapse. This suggests that better treatment outcomes may be achieved when patients remain in specialist care. This finding has important implications for the clinical setting and might provide insights on how to reduce relapse rates after antidepressant discontinuation overall in this patient population. One possible mechanism underlying this effect could be the impact of psychotherapy that is often delivered by specialists. Although in the current study psychotherapy did not appear to have an effect on relapse rates, our assessment of psychotherapeutic intervention strength was crude, and does leave room for the possibility that relapse risk could be mitigated by means of specific psychotherapeutic input. Indeed, psychotherapeutic techniques explicitly aimed at relapse have been developed^50,51^. Second, we did not replicate the effects of anxiety on relapse risk^26^, but the complete-case analyses replicated somatic pain as a risk factor^27^. Third, the null findings do replicate null findings from RCTs^21^ in a naturalistic setting. Importantly, the two indicators which clinical guidelines emphasize, namely the number of prior episodes and the length of ADM treatment^15,17^, both failed to show an association with relapse risk in our naturalistic setting. This mirrors previous findings in RCTs^21^ and the consistent lack of coherent effects of these measures on relapse risk after ADM discontinuation suggests a revisiting of these recommendations. In a similar vein, we found no effect of residual symptoms, a decision criterion added in the newest version of the guidelines^17^, on subsequent relapse risk. This is the case despite an influence of residual symptoms on overall relapse risk^20^ and symptom severity being the best predictor of disease course in studies using similar analyses approaches for patients in a depressive episode^47,49^. Finally, the lack of effects of any other clinical variable is still surprising given the relation to overall relapse risk of several of them as reviewed previously^52,53^.

Next, discontinuation was associated with a robust increase in current symptoms across domains. Surprisingly, this increase in symptoms did not appear to be related to prospective relapses. The dissociation we observed raises the possibility that the mechanisms driving symptom increase after discontinuation differ from those driving subsequent relapse even though relapse trajectories on and off medication are similar^54^. Clinically, the fact that transient symptomatic worsening does not relate to relapse may help clinicians and patients alike to hold their nerve in the face of early worsening of symptoms.

The study has strengths and limitations. Most prominently, the naturalistic setting of the study limits our ability to draw causal inferences: the pharmacological discontinuation effect is confounded with the potential psychological effect of knowing that the medication has been discontinued, and these cannot be disentangled. Additionally, predictors might differ between patients with a true-drug response and patients with placebo response, but our study design does not allow us to disentangle these two groups. As we excluded patients with other psychotropic medications, our results might not generalize to patients who are treated with other medication in parallel. However, the naturalistic design increases the relevance for real-life outpatient care where these effects co-occur. Furthermore, by including patients with comorbid anxiety disorders, we target the most prevalent population treated with antidepressant in primary care^19^. A further strength is the application of cross-validation to examine generalisability, but the small sample size is an important limitation. The small sample size also limits the identifiability of mechanistically heterogeneous subgroups and complicates the interpretation of null results. However, preparing an a priori analysis plan and carefully executing it adds additional credibility to our results. Based on the power of our study, the null results exclude large effects. In addition, using data from the same patient sample, we could show that decision time during a physical effort task predicted relapse better than chance in a validation dataset^55^ and that changes in resting state-functional connectivity between the right dorsolateral prefrontal cortex and the parietal cortex due to discontinuation predicted subsequent relapse using LOOCV^56^ . These results indicate that predictors of relapse can be identified and validated in our sample and that behavioural and biomarkers seem to carry more predictive weight for relapse after antidepressant discontinuation. We will continue to examine more potential predictors using behavioural, fMRI, EEG and physiological data from this sample.

### Clinical implications

The results of the present study need to be replicated. Nevertheless, they are of potential clinical relevance and suggest several changes to the management of remitted depressive disorders. First, there may be a role for continued specialist care, in particular during and after the discontinuation phase. Second, prominent decision criteria currently used in clinical practice such as length of treatment, number of prior episodes and residual symptoms are poorly predictive of relapse, suggesting that guidelines for antidepressant discontinuation might have to be revisited. Third, both treatment providers and patients need to be informed that discontinuation may be accompanied by a transient re-emergence of depressive symptoms that do not necessarily indicate an imminent relapse.

### Conclusion

Easily assessable demographic and clinical variables appear to be of limited use to guide antidepressant discontinuation decisions. Given the importance of the problem, more complex and costly measures should be evaluated.

## Supplementary Information

Supplementary Information.

## References

[1] WHO. *Depression and Other Common Mental Disorders: Global Health Estimates* (World Health Organization, 2017).

[2] Lépine J-P, Briley M (2011). The increasing burden of depression. Neuropsychiatr. Dis. Treat..

[3] American Psychiatric Association. *Diagnostic and Statistical Manual of Mental Disorders*, 4th edn. (American Psychiatric Association, 2000).

[4] Geddes JR (2003). Relapse prevention with antidepressant drug treatment in depressive disorders: A systematic review. Lancet.

[5] Kaymaz N, van Os J, Loonen AJM, Nolen WA (2008). Evidence that patients with single versus recurrent depressive episodes are differentially sensitive to treatment discontinuation: A meta-analysis of placebo-controlled randomized trials. J. Clin. Psychiatry.

[6] Glue P, Donovan MR, Kolluri S, Emir B (2010). Meta-analysis of relapse prevention antidepressant trials in depressive disorders. Aust. N. Z. J. Psychiatry.

[7] Sim K, Lau WK, Sim J, Sum MY, Baldessarini RJ (2015). Prevention of relapse and recurrence in adults with major depressive disorder: Systematic review and meta-analyses of controlled trials. Int. J. Neuropsychopharmacol..

[8] Rush AJ (2006). Acute and longer-term outcomes in depressed outpatients requiring one or several treatment steps: A STAR*D report. Am. J. Psychiatry.

[9] Olfson M, Marcus SC, Tedeschi M, Wan GJ (2006). Continuity of antidepressant treatment for adults with depression in the United States. Am. J. Psychiatry.

[10] Hunot VM, Horne R, Leese MN, Churchill RC (2007). A cohort study of adherence to antidepressants in primary care: The influence of antidepressant concerns and treatment preferences. Prim. Care Companion J. Clin. Psychiatry.

[11] Uher R (2010). Trajectories of change in depression severity during treatment with antidepressants. Psychol. Med..

[12] Gueorguieva R, Mallinckrodt C, Krystal JH (2011). Trajectories of depression severity in clinical trials of duloxetine: Insights into antidepressant and placebo responses. Arch. Gen. Psychiatry.

[13] Muthén B, Asparouhov T, Hunter AM, Leuchter AF (2011). Growth modeling with nonignorable dropout: Alternative analyses of the Star*D antidepressant trial. Psychol. Methods.

[14] Musliner KL (2016). Heterogeneity in 10-year course trajectories of moderate to severe major depressive disorder: A Danish national register-based study. JAMA Psychiatry.

[15] Bauer M (2013). World Federation of Societies of Biological Psychiatry (WFSBP) guidelines for biological treatment of unipolar depressive disorders, part 1: Update 2013 on the acute and continuation treatment of unipolar depressive disorders. World J. Biol. Psychiatry.

[16] NICE. *Depression: The Treatment and Management of Depression in Adults (Updated Edition).* (British Psychological Society, 2010). 22132433

[17] NICE. *Depression in Adults: Recognition and Management. Clinical Guideline [CG90]* (National Institute for Health and Care Excellence, 2019). https://www.nice.org.uk/guidance/cg90 31990491

[18] Frank, E. *et al.* Conceptualization and rationale for consensus definitions of terms in major depressive disorder. Remission, recovery, relapse, and recurrence. *Arch. Gen. Psychiatry* **48**, 851–855 (1991). 10.1001/archpsyc.1991.01810330075011 1929776

[19] Kessler RC (2003). The epidemiology of major depressive disorder: Results from the national comorbidity survey replication (NCS-R). JAMA.

[20] Nierenberg AA (2010). Residual symptoms after remission of major depressive disorder with citalopram and risk of relapse: A STAR*D report. Psychol. Med..

[21] Berwian IM, Walter H, Seifritz E, Huys QJM (2017). Predicting relapse after antidepressant withdrawal—A systematic review. Psychol. Med..

[22] Viguera AC, Baldessarini RJ, Friedberg J (1998). Discontinuing antidepressant treatment in major depression. Harv. Rev. Psychiatry.

[23] Andrews PW, Kornstein SG, Halberstadt LJ, Gardner CO, Neale MC (2011). Blue again: Perturbational effects of antidepressants suggest monoaminergic homeostasis in major depression. Front. Psychol..

[24] Trinh N-HT (2011). Examining the role of race and ethnicity in relapse rates of major depressive disorder. Compr. Psychiatry.

[25] McGrath PJ (2000). Predictors of relapse during fluoxetine continuation or maintenance treatment of major depression. J. Clin. Psychiatry.

[26] Joliat MJ (2004). Long-term treatment outcomes of depression with associated anxiety: Efficacy of continuation treatment with fluoxetine. J. Clin. Psychiatry.

[27] Fava M (2009). Predictors of relapse in a study of duloxetine treatment in patients with major depressive disorder. J. Affect. Disord..

[28] Stewart JW (1998). Use of pattern analysis to predict differential relapse of remitted patients with major depression during 1 year of treatment with fluoxetine or placebo. Arch. Gen. Psychiatry.

[29] Nierenberg AA, Quitkin FM, Kremer C, Keller MB, Thase ME (2004). Placebo-controlled continuation treatment with mirtazapine: Acute pattern of response predicts relapse. Neuropsychopharmacology.

[30] Quitkin, F. M. *et al.* Use of pattern analysis to identify true drug response. A replication. *Arch. Gen. Psychiatry* **44**, 259–264 (1987). 10.1001/archpsyc.1987.01800150071009 3548638

[31] Lo A, Chernoff H, Zheng T, Lo S-H (2015). Why significant variables aren’t automatically good predictors. Proc. Natl. Acad. Sci. U.S.A..

[32] Huys QJM, Maia TV, Frank MJ (2016). Computational psychiatry as a bridge from neuroscience to clinical applications. Nat. Neurosci..

[33] Wakefield JC, Schmitz MF (2013). When does depression become a disorder? Using recurrence rates to evaluate the validity of proposed changes in major depression diagnostic thresholds. World Psychiatry.

[34] Williams JB (1988). A structured interview guide for the Hamilton depression rating scale. Arch. Gen. Psychiatry.

[35] Wittchen H-U, Fydrich T (1997). Strukturiertes klinisches Interview für DSM-IV. Manual zum SKID-I und SKID-II.

[36] Zou, H. & Hastie, T. Regularization and variable selection via the elastic net. *J. R. Stat. Soc. Ser. B (Stat. Methodol.)* **67**, 301–320 (2005).

[37] Vittengl JR, Jha MK, Minhajuddin A, Thase ME, Jarrett RB (2021). Quality of life after response to acute-phase cognitive therapy for recurrent depression. J. Affect. Disord..

[38] Lehr S (2005). Mehrfachwahl-Wortschatz-Intelligenztest MWT-B.

[39] Rush AJ, Gullion CM, Basco MR, Jarrett RB, Trivedi MH (1996). The inventory of depressive symptomatology (IDS): Psychometric properties. Psychol. Med..

[40] Spitzer RL, Kroenke K, Williams JBW, Löwe B (2006). A brief measure for assessing generalized anxiety disorder: The GAD-7. Arch. Intern. Med..

[41] Derogatis LR, Cleary PA (1977). Confirmation of the dimensional structure of the SCL-90: A study in construct validity. J. Clin. Psychol..

[42] Hollon SD (2006). Presenting characteristics of depressed outpatients as a function of recurrence: Preliminary findings from the STAR*D clinical trial. J. Psychiatr. Res..

[43] Keller MB, Lavori PW, Lewis CE, Klerman GL (1983). Predictors of relapse in major depressive disorder. JAMA.

[44] Keller MB (1984). Long-term outcome of episodes of major depression: Clinical and public health significance. J. Am. Med. Assoc..

[45] Monroe SM, Harkness KL (2005). Life stress, the “kindling” hypothesis, and the recurrence of depression: Considerations from a life stress perspective. Psychol. Rev..

[46] Monroe SM, Harkness KL (2011). Recurrence in major depression: A conceptual analysis. Psychol. Rev..

[47] Chekroud AM (2016). Cross-trial prediction of treatment outcome in depression: A machine learning approach. Lancet Psychiatry.

[48] Wolfers T, Buitelaar JK, Beckmann CF, Franke B, Marquand AF (2015). From estimating activation locality to predicting disorder: A review of pattern recognition for neuroimaging-based psychiatric diagnostics. Neurosci. Biobehav. Rev..

[49] Dinga R (2018). Predicting the naturalistic course of depression from a wide range of clinical, psychological, and biological data: A machine learning approach. Transl. Psychiatry.

[50] Hollon SD (2005). Prevention of relapse following cognitive therapy vs medications in moderate to severe depression. Arch. Gen. Psychiatry.

[51] Hollon SD (2014). Effect of cognitive therapy with antidepressant medications vs antidepressants alone on the rate of recovery in major depressive disorder: a randomized clinical trial. JAMA Psychiatry.

[52] Burcusa SL, Iacono WG (2007). Risk for recurrence in depression. Clin. Psychol. Rev..

[53] Hardeveld F, Spijker J, De Graaf R, Nolen WA, Beekman ATF (2010). Prevalence and predictors of recurrence of major depressive disorder in the adult population. Acta Psychiatr. Scand..

[54] Gueorguieva R, Chekroud AM, Krystal JH (2017). Trajectories of relapse in randomised, placebo-controlled trials of treatment discontinuation in major depressive disorder: An individual patient-level data meta-analysis. Lancet Psychiatry.

[55] Berwian IM (2020). Computational mechanisms of effort and reward decisions in patients with depression and their association with relapse after antidepressant discontinuation. JAMA Psychiatry.

[56] Berwian IM (2020). The relationship between resting-state functional connectivity, antidepressant discontinuation and depression relapse. Sci. Rep..

