# Supplementary material for: Low predictive power of clinical features for relapse prediction after antidepressant discontinuation in a naturalistic setting
Source: Sci Rep. 2022 Jul 1;12:11171. doi: 10.1038/s41598-022-13893-9 (PMC9249776; doi:10.1038/s41598-022-13893-9)
Supplement: Supplementary file 1 — Supplementary Information. [file 41598_2022_13893_MOESM1_ESM.pdf]

Supplementary Material

Low predictive power of clinical features for relapse prediction after antidepressant discontinuation in a naturalistic setting

Isabel M. Berwian, Julia G. Wenzel, Leonie Kuehn, Inga Schnuerer, Erich Seifritz, Klaas E. Stephan, Henrik Walter and Quentin J. M. Huys

S1 Supplementary Methods

S1.1 In- and Exclusion Criteria

Participants fulfilling the following inclusion criteria were eligible for participation in the study:

1. age 18-55 years
2. ability to consent and adhere to the study protocol
3. written informed consent
4. fluent in written and spoken German.

Patients had to additionally fulfil the following criteria:

1. currently under medical care with a psychiatrist or general practitioner for remitted Major Depressive Disorder and willing to remain in care for the duration of the study (approx. 9 months)
2. informed choice to discontinue medication (including willingness to taper the medication over at most 12 weeks) that was independent of study participation
3. clinical remission ($HAMD_{17}$ of less than 7) had been achieved under therapy with Antidepressant Medication (ADM) without having undergone manualized psychotherapy (as reported by treating clinicians); with no other concurrent psychotropic medication and had been maintained for a minimum of 30 days,
4. consent to information exchange between treating physician and study team members regarding inclusion/exclusion criteria and past medical history.

Any of the following exclusion criteria led to exclusion of an participant. This included the following general criteria

1. any disease of type and severity sufficient to influence the planned measurement or to interfere with the parameters of interest (This includes neurological, endocrinological, oncological comorbidities, a history of traumatic or other brain injury, neurosurgery or longer loss of consciousness.)
2. premenstrual syndrome (ICD-10 N94.3).

and MRI-related criteria

1. MRI-incompatible metal parts in the body,
2. inability to sit or lie still for a longer period,
3. possibility of presence of any metal fragments in the body,
4. pregnancy,
5. pacemaker, neurostimulator or any other head or heart implants,
6. claustrophobia and
7. dependence on hearing aid.

For patients the following additional criteria would led to exclusion:

1. current psychotropic medication other than antidepressants,
2. questionable history of major depressive episodes without complicating factors,
3. current acute suicidality,

4. lifetime or current axis II diagnosis of borderline or antisocial personality disorder,
5. lifetime or current psychotic disorder of any kind, bipolar disorder,
6. current posttraumatic stress disorder, obsessive compulsive disorder, or eating disorder
7. current drug use disorder (with the exception of nicotine) or within the past 5 years.

Healthy controls were excluded if there was a lifetime history of Diagnostic and Statistical Manual of Mental Disorders (4th ed., text rev.; DSM-IV-TR; 1) axis I or axis II disorder with the exception of nicotine dependence.

S1.2 Procedures

Details for all interviews and questionnaires relevant for the present study are described in section S1.3.

S1.2.1 Recruitment

Participants were recruited using two types of strategies: 1) we informed outpatient clinics, practicing psychiatrist and general practitioners about the study with help of presentations and letters and distributed leaflets requesting them to inform potentially eligible patients about the option to participate in the study; 2) we recruited patients directly from the general population sending emails to staff and students of the universities, distributing leaflets to households, advertising in newspapers, streetcars and Google, and publicity about the study in newspaper articles. It might be of note for researcher planning future studies with a similr population, that we recruited a large majority of patients via the second strategy.

S1.2.2 Randomisation

After inclusion, participants were openly randomised into one of the two study arms, i.e. they either discontinued before the second main assessment (MA2; group 1D2) or did both MAs before discontinuation (group 12D). We assumed *a priori* that severity might affect relapse rates. Hence, to ensure that both groups would contain a sample with equal distributions of severity, we stratified patients into a severe and a non-severe group. If patients fulfilled either of the following criteria, they were assigned to the severe group: 1) more than three prior episodes, 2) more than seven depressive symptoms during the last episode, 3) severely impaired social functions, i.e. strongly isolated, disabled or aggressive, 4) engagement in activities almost nonexistent, 5) capacity to work almost nonexistent. We, additionally, stratified for site. Group membership was allocated by a randomisation algorithm. The first ten subjects at each site were not randomised, but all assigned to arm 1D2.

S1.2.3 Baseline Assessment

Participants who passed an initial telephone screening were invited for an on-site baseline assessment (BA). The first part of the BA was designed to assess if participants were eligible for the study. Inclusion criteria are listed in section S1.1. Participants underwent three clinical interviews to assess 1) current depressive symptoms, 2) axis-I disorders and 3) axis-2 disorders, i.e. personality disorders. Furthermore, we conducted a life-long medication screening on pharmaceuticals, a questionnaire to check for eligibility to enter a magnetic resonance imaging scanner, as well as individual questions relating to treatment. Participants had to sign that they discontinue their medication voluntarily, be in treatment with a physician who agreed to accompany the discontinuation process and wave the medical privilege of that physician with regard to the study team. If inclusion criteria were met, participants were randomised to either study arm, filled out a questionnaire batch assessing stable traits and underwent a short neuropsychological testing.

S1.2.4 Main Assessments

During both main assessments participants underwent four types of assessments: 1) an fMRI session including tasks to probe affective decision-making, automatic and voluntary emotion regulation, memory and a resting-state session, 2) a venipuncture to assess genetics (MA1 only), epigenetics, gene expression, cytokines, brain-derived neural growth factor, pharmacology level (before discontinuation only), C-reactive protein and a blood count, 3) behavioural testing including behavioural task to measure effort-related decision-making, impulsivity and emotion recognition and another questionnaire batch assessing state variables and 4) a questionnaire batch assessing state variables. In addition, current depressive symptoms were assessed in a clinical interview. In the

two mornings before MA1, participants were asked to collect saliva tubes such that morning cortisol levels could be derived. Patients randomised to group 12D were asked to come for a third visit after discontinuation to undergo an additional venipuncture.

On top of these assessments, participants at the Zurich side were offered the option of additionally taking part in an electroenecelography (EEG) session including tasks measuring emotional reactivity, loudness dependent auditory potential, vigilance, resting-state and heartbeat evoked potentials.

S1.2.5 Follow-up Period and Final Assessment

During the follow-up period, patients were regularly contacted by phone and had to fill out short online questionnaire batches to assess symptom changes. Final assessment (FA) took either place if patients had a relapse or after six months. Current depressive symptoms were assessed and the state questionnaire batch was repeated.

S1.3 Questionnaires and Clinical Assessments

Clinical in- and exclusion criteria, as well as disease history and course, were assessed with the Structured Clinical Interview for DSM-IV (SCID) I and II to diagnose axis 1 disorders (major mental disorders) and axis II disorders (personality disorders), respectively (7). The Structured Interview Guide for Hamilton Depression Rating Scale (SIGH-D; 6) consisting of 17 items was used to assess inclusion and the Inventory of Depressive Symptomatology Clinician Rated (IDS-C; 4) with 30 items to quantify residual depression. Intelligence was assessed with the Mehrfachwahl Wortschatz Test (3). The symptom checklist (SCL-90; 2) measures psychological impairment using nine subscales with 83 items in total and seven additional items. The global severity index (GSI) is the mean of all responses and is used to assess general psychological impairment. To assess somatic pain, we used the average score of somatisation subscale. This subscale contains twelve items and reflects simple somatic burden and functional disorder. We used a 7-item scale developed to screen for generalised anxiety disorder (GAD-7 5) to measure residual anxiety symptoms.

Additionally, we included questionnaires in the study but not in the reported analyses that tapped into the following domains: anhedonia/motivation, anxiety, mood, sexuality, discontinuation symptoms, personality, optimism/pessimism, emotion regulation, thinking style, resilience, self control, stressor load, quality of life, trauma, and demography. Questionnaires measuring stable traits and past experiences were assessed during BA. Measures assumed to differ within short time periods and be affected by discontinuation and disease state were assessed at MA1, MA2 and FA.

S1.4 Data Analysis

Disease severity corresponds to the first principal component of a principle component analysis including the variables number of past depressive episodes, age at illness onset, time in remission, time since depression onset, severity of last episode, time sick in total and time sick in the last five year as variables.

67% of patients took a selective serotonin reuptake inhibitor, 27% a serotonin-norepinephrine reuptake inhibitor and 7% an antidepressant from a different class. Medication load was based on the dose prior to discontinuation divided by the maximal allowed dose according to the Swiss compendium (www.compendium.ch) and by the weight of the participant.

Psychotherapy score was coded such that patients with no psychotherapy within the year before the study received a 0, patients reporting to have completed a psychotherapy within one year before the study a 0.5 and patients reporting to be in psychotherapy at the beginning of the study as 1. Significance was computed with a three-way chi-squared test.

For duration of antidepressant medication intake is the median and the interquartile range are reported. Significance was computed by means of the rank sum test. Duration of antidepressant intake was measured in months.

S2 Supplementary Results

Figure S1 shows reasons for dropouts in the patient sample.

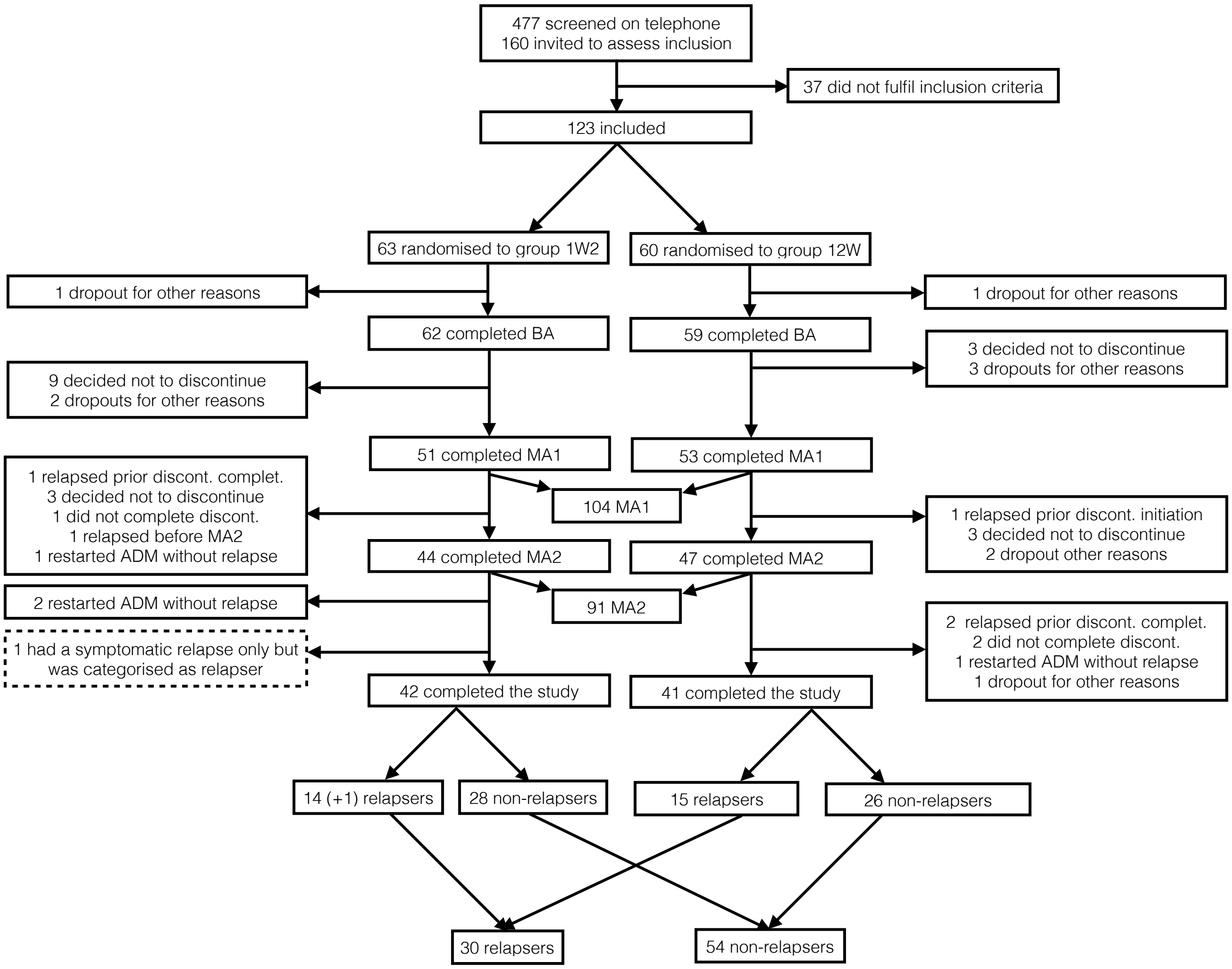

Figure S1: Consort Diagram: Depicted are reasons for dropouts and exclusion for patients throughout the study. (+ X) indicates the number of participants who relapsed after discontinuation but before MA2.

Patients who did not complete the study had more residual symptoms ($t(102) = -2.01$, $p = 0.047$, CI = -3.74 - -0.02) and more symptoms during the last episode ($t(102) = -2.09$, $p = 0.039$, CI = -1.24 - -0.03) as shown in Table S1.

Table S2 depicts details results of the discontinuation effect, the discontinuation relapse interaction effect and test-retest reliability for residual depression, residual anxiety, somatic pain and general impairment.

Table S1: Dropout Comparisons

	Study completed (n = 84)	Dropouts (n = 20)	p value
**Demographics**			
Age	34.79 (11.19)	35.10 (11.02)	0.91
Male sex, No. (%)	20 (24)	4 (20)	0.72
Intelligence[b]	28.40 (4.40)	26.9 (4.49)	0.17
Site Berlin, No. (%)	23 (27)	5 (25)	0.83
**Clinical predictors**			
Current symptoms			
Residual depression[b]	3.37 (3.73)	5.25 (3.88)	0.047
Residual anxiety[b]	2.73 (2.41)	3.60 (1.90)	0.13
Somatic pain[b]	0.32 (0.23)	0.35 (0.24)	0.63
General impairment[b]	0.29 (0.24)	0.39 (0.25)	0.09
Clinical history			
Age of onset	24.23 (8.88)	22.55 (9.60)	0.46
Chronicity[b]	8.12 (9.73)	10.00 (11.05)	0.45
Severity[b]	7.01 (1.24)	7.65 (1.18)	0.039
Number of prior episodes	2.45 (1.61)	2.50 (1.67)	0.91
Disease severity[c]	0.02 (0.35)	0.05 (0.48)	0.74
Comorbidities[b]	0.76 (1.12)	1.30 (1.26)	0.06
Treatment			
Treated by GP only, No. (%)	18 (21)	4 (20)	0.89
Length of ADM intake[c]	23.5 (36)	21.5 (22.5)	0.16
Medication load[c]	0.01 (0.004)	0.01 (0.004)	0.32
Psychotherapy[c]	0.39 (0.39)	0.38 (0.39)	0.99

a) Unless stated otherwise, mean (SD) are shown; b) Determined as follows: intelligence: Mehrfachwahl Wortschatz Test (3); residual depression: Inventory of Depressive Symptomatology-Clinician Rated (4); residual anxiety: screening generalised anxiety disorder (GAD-7; 5); somatic pain: somatisation subscale of symptom checklist 90 (SCL-90; 2); general impairment: global severity index of SCL-90; chronicity: numbers of months sick within the last 5 years; severity: symptoms during the last episode; comorbidities: number of past and present psychiatric diagnoses c) Computation of the variables is described in the section S1.4. GP = general practitioner

Table S2: Discontinuation effects

Changes from MA1 to MA2 in all patients								
	Discontinuation			No discontinuation			Discontinuation vs. No discont.	
	MA1	MA2	p value	MA1	MA2	p value	p value (MA1)	p value (MA2)
Residual depression	3.39 (4.19)	7.64 (5.69)	<0.001	3.64 (3.39)	4.64 (3.40)	0.11	0.75	0.006
Residual anxiety	2.73 (2.42)	4.02 (2.81)	0.009	2.68 (2.35)	2.43 (2.40)	0.73	0.93	0.009
Somatic pain	0.33 (0.23)	0.48 (0.40)	0.02	0.31 (0.22)	0.34 (0.26)	0.44	0.69	0.12
General impairment	0.29 (0.23)	0.38 (0.23)	0.03	0.30 (0.26)	0.24 (0.22)	0.10	0.85	0.02
Changes from MA1 to MA2 in patients who discontinued								
	Relapsers			Non-relapsers			Relapsers vs. Non-relapsers	
	MA1	MA2	p value	MA1	MA2	p value	p value (MA1)	p value (MA2)
Residual depression	4.43 (6.58)	10.64 (7.07)	0.014	2.68 (2.34)	6.04 (4.44)	<0.001	0.21	0.02
Residual anxiety	3.79 (3.19)	5.64 (2.79)	0.09	2.21 (1.85)	3.25 (2.61)	0.07	0.07	0.04
Somatic pain	0.43 (0.26)	0.62 (0.47)	0.12	0.26 (0.20)	0.41 (0.34)	0.04	0.05	0.12
General impairment	0.39 (0.29)	0.51 (0.20)	0.14	0.23 (0.18)	0.30 (0.20)	0.11	0.07	0.01
Changes from MA1 to MA2 in patients who did not discontinue								
	Relapsers			Non-relapsers			Relapsers vs. Non-relapsers	
	MA1	MA2	p value	MA1	MA2	p value	p value (MA1)	p value (MA2)
Residual depression	3.13 (3.95)	4.40 (3.11)	0.60	3.65 (1.80)	4.58 (3.6)	0.60	0.83	0.88
Residual anxiety	2.20 (2.81)	2.27 (1.75)	0.95	2.92 (2.13)	2.89 (2.81)	0.95	0.89	0.89
Somatic pain	0.37 (0.21)	0.33 (0.21)	0.70	0.30 (0.25)	0.37 (0.28)	0.70	0.70	0.70
General impairment	0.31 (0.35)	0.26 (0.17)	0.79	0.27 (0.20)	0.24 (0.25)	0.79	0.79	0.79

Variables were measured with the following instruments: residual depression: Inventory of Depressive Symptomatology-Clinician Rated (4); residual anxiety: screening generalised anxiety disorder (GAD-7; 5); somatic pain: somatisation subscale of symptom checklist 90 (SCL-90; 2); general impairment: global severity index of SCL-90. All p-values are corrected according to false-discovery-rate. MA1 = main assessment 1; MA2 = main assessment 2;

References

[1] **American Psychiatric Association** (2000). *Diagnostic and Statistical Manual of Mental Disorders 4th ed., text rev.* Author, Washington, DC.

[2] **Derogatis LR**, **Cleary PA** (1977). Confirmation of the dimensional structure of the SCL-90: a study in construct validity. *Journal of Clinical Psychology* **33**(4), 981–989.

[3] **Lehr S** (2005). *Mehrfachwahl-Wortschatz-Intelligenztest MWT-B*. Spitta, Balingen, DE.

[4] **Rush AJ**, **Gullion CM**, **Basco MR**, **Jarrett RB**, **Trivedi MH** (1996). The inventory of depressive symptomatology (IDS): psychometric properties. *Psychol Med* **26**(3), 477–86.

[5] **Spitzer RL**, **Kroenke K**, **Williams JBW**, **Löwe B** (2006). A brief measure for assessing generalized anxiety disorder: the GAD-7. *Arch Intern Med* **166**(10), 1092–7.

[6] **Williams JB** (1988). A structured interview guide for the hamilton depression rating scale. *Arch Gen Psychiatry* **45**(8), 742–7.

[7] **Wittchen HU**, **Fydrich T** (1997). *Strukturiertes klinisches Interview für DSM-IV. Manual zum SKID-I und SKID-II*. Hofgrefe, Göttingen, DE.
